# Feature Selection and Comparison of Machine Learning Algorithms in Classification of Grazing and Rumination Behaviour in Sheep

**DOI:** 10.3390/s18103532

**Published:** 2018-10-19

**Authors:** Nicola Mansbridge, Jurgen Mitsch, Nicola Bollard, Keith Ellis, Giuliana G. Miguel-Pacheco, Tania Dottorini, Jasmeet Kaler

**Affiliations:** 1 School of Veterinary Medicine and Science, University of Nottingham, Sutton Bonington Campus, Leicestershire LE12 5RD, UK; svyncm@exmail.nottingham.ac.uk (N.M.); nicola.bollard@nottingham.ac.uk (N.B.); giuliana.miguelpacheco1@nottingham.ac.uk (G.G.M.-P.); Tania.Dottorini@nottingham.ac.uk (T.D.); 2 School of Computer Science, Jubilee Campus, University of Nottingham, Nottingham NG8 1BB, UK; Jurgen.Mitsch@nottingham.ac.uk; 3 Advanced Data Analysis Centre, University of Nottingham, Nottingham NG8 1BB, UK; 4 Internet of Things Systems Research, Intel Labs, Leixlip W23 CX68, Ireland; keith.a.ellis@intel.com

**Keywords:** sheep behaviour, grazing, rumination behaviour, classification algorithm, accelerometer and gyroscope, sensor, machine learning, precision livestock monitoring

## Abstract

Grazing and ruminating are the most important behaviours for ruminants, as they spend most of their daily time budget performing these. Continuous surveillance of eating behaviour is an important means for monitoring ruminant health, productivity and welfare. However, surveillance performed by human operators is prone to human variance, time-consuming and costly, especially on animals kept at pasture or free-ranging. The use of sensors to automatically acquire data, and software to classify and identify behaviours, offers significant potential in addressing such issues. In this work, data collected from sheep by means of an accelerometer/gyroscope sensor attached to the ear and collar, sampled at 16 Hz, were used to develop classifiers for grazing and ruminating behaviour using various machine learning algorithms: random forest (RF), support vector machine (SVM), *k* nearest neighbour (kNN) and adaptive boosting (Adaboost). Multiple features extracted from the signals were ranked on their importance for classification. Several performance indicators were considered when comparing classifiers as a function of algorithm used, sensor localisation and number of used features. Random forest yielded the highest overall accuracies: 92% for collar and 91% for ear. Gyroscope-based features were shown to have the greatest relative importance for eating behaviours. The optimum number of feature characteristics to be incorporated into the model was 39, from both ear and collar data. The findings suggest that one can successfully classify eating behaviours in sheep with very high accuracy; this could be used to develop a device for automatic monitoring of feed intake in the sheep sector to monitor health and welfare.

## 1. Introduction

Grazing and ruminating are the most important behaviours for ruminants and are how they spent most of their time [1]. Monitoring these behaviours for extensive livestock, especially sheep, offers an effective way to understand grazing patterns, monitor animal health and welfare and understand utilisation of forage by the flock [2].

Continuous monitoring of eating behaviour performed by human operators has many difficulties. Large numbers of animals are often involved, making manual monitoring time-consuming and labour-intensive [3]. The expansion of commercial farming, with higher animal numbers and a focus on success of the business, results in a reduction in time and labour dedicated to animal assessment. This problem is exacerbated when animals are kept at pasture or are free-ranging [2]. Another problem with manual observation is the necessity of having an observer present to do the monitoring; animals may at times not behave normally when a human is present, having a detrimental effect upon the accuracy of any results [4]. For these reasons, the development of an automatic recording device to monitor animal behaviour would be a useful tool in terms of animal health, welfare, productivity and resource efficiency [5].

In recent years, accelerometers and gyroscopes have been used for behaviour identification due to their relevance and potential applications [3,6,7,8]. However, the majority of research surrounding the automatic monitoring of livestock behaviour in general, including eating behaviour, has been on dairy cattle with high reported accuracies around 84–96% for various eating behaviours [3,9,10,11]. There are some commercial monitoring systems that are available for dairy cattle that are used to capture feeding behaviours such as Lely [12] and MooMonitors [13]. However, none are available for sheep. The automatic systems for cattle cannot be directly applied to sheep as there is difference in accelerometer signal patterns between species [14], which means that different algorithms are needed. Moreover, a different form factor is needed for the hardware, due to difference in size and weight requirements. 

In comparison to dairy cattle, there are far fewer studies classifying eating behaviour of sheep. The accuracies of classification reported by these studies are quite high (>90% [2,5,15,16,17]). However, all the studies that have been done so far have used very few data points for algorithmic classification [2,5,15,18,19] or have used largely unbalanced datasets [2]. The latter issue in particular can potentially lead to overfitting and inflated accuracies [20]. In addition, none of the aforementioned studies have investigated the classification of all the eating behaviours (i.e., grazing and ruminating); focusing only on grazing [2,19]. However, there is evidence from work in cattle that certain health events are only reflected in certain types of eating behaviour. For example, a recent study identified that lameness in cows only affects feeding (total duration and frequency) behaviour and not ruminating behaviour [21], suggesting that there is value in differentiating these behaviours in classification.

To develop a real world practical and useful solution, it is important to evaluate various placements for sensors, as well as the most energy efficient way of sampling and processing the data (i.e., choosing the sampling rate and size of the time window for feature extraction). None of the previous studies evaluated different positions of sensors in terms of accuracy of classification for eating behaviour in sheep and used sampling rate of 20–25 Hz [2]. Position and sampling rates could impact the accuracy of the algorithms, with higher sampling rate resulting in higher power consumption [22]. In our previous work we demonstrated that when using an accelerometer and gyroscope sensor the optimum sampling rate needed to classify lying, standing and walking behaviour in sheep with respect to accuracy and energy efficiency is 16 Hz with a 7-s sample window [16].

The aims of this study were as follows: (a) classify grazing and ruminating behaviour in sheep using various machine learning algorithms (b) determine the optimal number of features that should be extracted from the data and used for classification, (c) compare multiple algorithms (random forest, support vector machine, *k* nearest neighbour and adaptive boost) in terms of several classification performance indicators and (d) investigate classification performance as a function of placement on ear and collar. In all cases an accelerometer and gyroscope sensor, sampling at 16 Hz was to be used.

## 2. Materials and Methods

### 2.1. Study Site and Animals

Initially, a pilot study was conducted over two days to validate the research protocols described below. Ethical permission was obtained from School of Veterinary Medicine and Science, University of Nottingham (Approval Number: 1481 150603). The main trial that followed the pilot study was conducted over a period of eight days: 5–7 October 2016 and 10–14 October 2016. A total of six sheep were selected (stratified random sampling regarding age) from a flock of 140 animals at the University of Nottingham. Characteristics such as body condition score, age and breed were assessed on the first day of data collection. Body condition scoring of sheep is used to assess the degree of fatness and body condition of the living animal. For scoring, U.K. industry guidelines were applied [23,24]. Body condition scores in the selected sheep ranged from 2.5 to 4. Ages ranged from 18 months to four years old. Sheep breeds included Texel cross (three individuals), Suffolk cross (one individual) and Mule (two individuals). During the day, when recordings were taking place, sheep were kept in a rectangular, 0.3-acre field with a 179.3 m perimeter. At night, sheep were allowed into a larger 2.1-acre field until the following morning when recording started again. For the duration of the observational study, these sheep were in a single group of 20 sheep. Sheep were marked with a number ranging from 1 to 19 to avoid confusion during the video analysis process; this was done using coloured livestock spray on either side to facilitate identification of individual sheep. Numbers sprayed on the sheep were re-sprayed again on the fifth day of the trial to ensure that they remained clearly legible throughout the entire study period.

### 2.2. Data Collection

Sensor data were collected using a custom-made wearable device based on the Intel^®^ Quark™ SE microcontroller C1000. The device encompassed a flash memory, a low power wide area radio module and a Bosch BMI160 (Bosch-sensortec.com, 2016) low power inertial measurement unit (IMU), featuring a 16-bit triaxial gyroscope and a 16-bit triaxial accelerometer. The devices were of dimension 31.6 × 35 × 9 mm and weighed 4 g. The devices were attached to a light-weight Li-Po battery 270mAh Li-ion battery.

**Figure 1:**
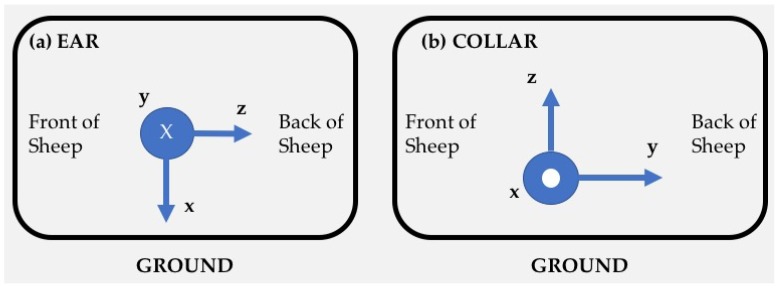
Sensor orientations for (**a**) ear and (**b**) collar mount configurations.

The devices were attached to the six sheep at two locations (a) to the existing electronic identification ear tag via a tape and lightweight plastic tie and (b) to neck collar using tape and lightweight plastic tie. All ear-mounted devices were fixed using the orientation illustrated in Figure 1 (left), whereas all collar-mounted devices were fixed using the orientation shown in Figure 1 (right). 

Devices were mounted on sheep at the beginning of each trial day at approximately 9 a.m. and were removed the following morning at 9 a.m., with the exception of 7 October 2016, when the devices were removed at 4 p.m. to prevent any possible damage over the weekend when no camera recording took place.

Each day, sensors were prepared by first setting the sampling frequency, then switching them on, whilst annotating the switching time. 

The sheep were also video recorded, as illustrated in Section 2.3. Therefore, to allow time synchronisation with the videos, sensors were shaken for 30 s and the start time of the shaking was annotated. This was followed by a procedure to establish a time reference, where sensors were held horizontally for 30 s and finally held vertically for 30 s. At the end of the 30 s of holding them vertically, the time was annotated. After this procedure, sensors were mounted on the sheep. The recorded data were downloaded from the devices after each period of operation. This process was later replaced in further trials with synchronisation handled over the radio. 

After removing spurious datasets due to sensor malfunction and data mishandling, 14 datasets with a total of 27,317 data points were used for analysis, with seven collected from ear sensors and seven from collar sensors.

### 2.3. Behavioural Observations

Sheep behavioural activities were recorded using a handheld Panasonic HC-V380 video camera with a tripod and were time stamped. The video camera was fitted with a 64 GB SanDisk elite SDXC UHS-1SD card to store the footage. The video camera was set to record in a MP4 50 M format with 1080p (1920 × 1080 pixels) quality. Video footage was recorded each day in the morning with duration of approximately 2 h. In the recording sessions, the starting and ending times of the recordings were annotated.

### 2.4. Behaviour Annotation of the Videos

Time stamped video recordings of the sheep were processed using the Noldus Observer XT 11 (Noldus) (www.noldus.com) software. Coding of the video recordings into the different behavioural categories or classes was performed by playing each video and manually pressing the corresponding code key of the identified behaviour from the set of predefined ones. Behaviours were defined based on the behaviour ethogram developed in a pilot study where sheep were observed and other literature [2]. Behaviours of interest for this study can be identified according to Table 1.

### 2.5. Data Processing

Processing of the data was performed using dedicated software written in Python 3.5 [25], specifically for this project. First, the raw sensor data (accelerometer and gyroscope) and the behaviour information from the video transcripts were aligned using the time stamps. Afterwards each file was discretised (converting continuous time series into discrete quantities [26]) into windows of equal length with a 50% overlap between two consecutive windows [18,19]. A 7 s window discretisation was used based on our previous work [16]. During coding of the video recording, an individual class label was assigned to each individual data sample. Therefore, the class discretisation of each window was determined by looking at the class labels of the individual data samples within each window. If all data samples within a window shared the same activity class, the collective label for the entire window was set to that particular activity class. Windows that contained data points with more than one activity class label were labelled as ’mixed’ windows and the predominant label was used as the class. The percentage of samples for the ‘mixed’ and ‘non-mixed’ windows in each of the sampling frequencies and window sizes in this study, is shown in Table 2.

For each time window, a set of feature characteristics [27] was extracted from the magnitude of the acceleration and the magnitude of the gyroscope, which are defined as follows:

(1) $$\;\bar{A}=\sqrt{A_{x}^{2}+A_{y}^{2}+A_{z}^{2}}\;$$

(2) $$\bar{G}=\sqrt{G_{x}^{2}+G_{y}^{2}+G_{z}^{2}},$$

where *Ax*, *Ay*, *Az*, *Gx*, *Gy*, *Gz* represent the acceleration and gyroscope signals at the axes *x*, *y*, *z*, respectively.

A total of 11 different feature characteristics were extracted from both the magnitude of the acceleration and from the magnitude of the gyroscope based on the previous literature and our work [16,28,29]. In addition, the same feature characteristics were computed from the rate of change of the magnitude of the acceleration (time derivative of the accelerometer signal) and from rate of change of the magnitude of the gyroscope (time derivative of the gyroscope signal), yielding a total of 44 features that were used in the classification. Extracted features include mean, standard deviation, kurtosis, minimum and maximum value [3], interquartile range [30], signal area, absolute signal area, number of zero crossings, dominant frequency [29] and spectral entropy [31].

### 2.6. Feature Selection

Feature selection in this study was carried out using a filter-based approach utilising ReliefF [32], which is an extension of the original Relief algorithm [33]. The key idea of all Relief-based algorithms is to estimate the quality of a feature by how well they distinguish between data samples that are close to each other. Weights assigned to the features represent their relevance to the classification problem. The ReliefF algorithm extends this idea for classification problems with more than two classes and incomplete datasets and has proven to be more robust than the original Relief algorithm [32]. Feature quality is assessed by looking at a randomly selected sample and its *k* nearest neighbours from the same class, called nearest hits, as well as its *k* nearest neighbours from each of the other classes, called nearest misses. Feature weights are then updated in an iterative process, depending on the values of nearest hits and nearest misses of each feature. The number of neighbours to consider is a user defined parameter (*n* = 100) and controls the locality of the estimates.

### 2.7. Classification Algorithms

Python 3.5 was used to develop eating behaviour classification models based on the features described in the previous section. Classification algorithm examined in this study include random forest (RF), support vector machine (SVM), *k* nearest neighbour (kNN) and adaptive boost (AdaBoost).

Random Forests [34,35] are a type of ensemble learning method that is formed through the combination of multiple decision trees trained on the training set. When applied to the test dataset, the predictions of the individual tree models within the random forest are combined into an overall classification decision, for example through means of a majority vote or through the application of weights. Because of this, random forest models correct overfitting (if present) due to the training set and provide robust classification performances [36].

A support vector machine is a type of non-probabilistic classifier that maps its inputs (i.e., the classification features) into a high-dimensional feature space, where each dimension represents one of the classification features. Support vector machines try to create a linear partition of the high-dimensional feature space into two subspaces. New, unseen data samples are later evaluated against this partition to determine their class membership. This method is non-probabilistic, because the features in the unseen data samples fully determine its location in the feature space of the support vector machine model [37]. 

kNN is a non-parametric, instance-based classification algorithm. In order to determine class membership, the algorithm takes the *k* closest samples from the training data as inputs. The most common class membership among the neighbours is then assigned to the object that is to be classified. Often the distances between the object of interest and its neighbours is weighted, so that nearer neighbours contribute more to the class membership majority vote than the more distant ones [38]. 

Adaptive boosting is an ensemble learning methodology that combines the output of several individual lower-level machine learners by majority vote to determine the final output of the ensemble classifier. Boosting is a step-wise procedure whereby a model is trained at each step. At every step, the weights assigned to the training samples are modified for the next step in a way so that previously misclassified training samples have their weights increased, whereas correctly classified samples have their weights decreased. AdaBoost supports a wide range of machine learning algorithms as base learners, but decision trees have proven to be a reliable and easy to use choice in the past [39,40].

### 2.8. Performance of the Classification

The performance of the classification algorithms was evaluated using the metrics of accuracy, precision, recall (also known as sensitivity), F-score and specificity, which can be computed as

(3) $$\;Accuracy=\frac{TP+TN}{TP+TN+FP+FN}\;$$

(4) $$\;Precision=\frac{TP}{TP+FP}\;$$

(5) $$\;Recall=\frac{TP}{TP+FN}\;$$

(6) $$\;F-Score=2\cdot \frac{Precision\cdot Recall}{Precision+Recall}\;$$

(7) $$Specificity=\frac{TN}{TN+FP},$$

where *TP* (true positives) is the number of instances where a behaviour was correctly classified as the behaviour that was observed (ground truth). *FN* (false negatives) is the number of instances where a particular behaviour was observed (ground truth) but misclassified by the algorithm as some other behaviour. *FP* (false positives) is the number of instances where the algorithm falsely classified a behaviour that was not observed. *TN* (true negative) is the number of instances where a behaviour was correctly classified as not being observed.

### 2.9. Precision, Recall, F-Score and Specificity

Whilst accuracy gives an overall measure of a classifier performance, measures such as precision, recall, F-score and specificity allow for a more detailed comparison of classification performance across different behaviours. These metrics are computed from the confusion matrix. In this type of matrix, each column represents the label predicted by the classifier whilst each row represents the observed label (ground truth). 

The overall accuracy represents the total number of correct classifications across all behaviours. This can be useful when it is equally important to correctly classify each behaviour. On the contrary, if one or more behaviours are of particular interest to the observer (e.g., grazing or ruminating), and the priority is to correctly classify them (possibly at the cost of achieving worse performance for the other behaviours), then precision, recall and specificity give a more adequate representation of the classifier performance. Finally, the F-score is calculated as the harmonic mean of precision and recall and combines precision and recall into one numerical measure. It reaches its best score at 1 and worst score at 0 [41].

### 2.10. Cross Validation

In this study, cross-validation was applied to evaluate the performance of each classification model, by repeatedly splitting the original dataset into training and testing subsets [42]. The 10-fold cross-validation was utilised as 10 has been shown to be a good and reasonable compromise between providing robust performance estimates and being computationally feasible, because the computational resource and time required increase with the number of iterations [42]. Different folds were tried to check the robustness of the predictions; however, there was no change in results.

To apply the 10-fold cross-validation the dataset was split into 10 subsets of equal size. Then, over a total of 10 iterations, one of the subsets was held back as a test set, whilst the remaining nine were used to train the classification model. The model was then evaluated using the test set. The process was repeated 10 times, so that each subset was used once as a testing set. This yielded 10 sets of performance values, and their average represented the cross-validated performance estimates for the classification model. Stratification was applied when partitioning the dataset into the 10 subsets, to ensure that the class representations in each of the subsets was equal to the full dataset [43].

## 3. Results

### 3.1. Feature Selection

**Figure 2:**
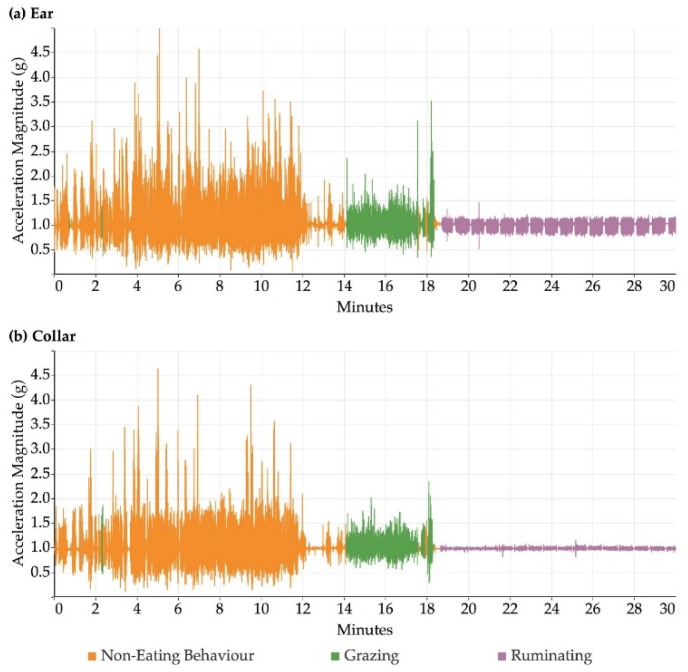
Example graph showing accelerometer data extracted from sensors mounted on one sheep in two locations, ear (**a**) and collar (**b**), over 30 min showing grazing, non-eating and ruminating behaviour over time.

Figure 2 illustrates an example time series of the accelerometer magnitude output for observed periods of grazing, ruminating and non-eating behaviour recorded by the ear sensor (Figure 2, top) and by the collar sensor (Figure 2, bottom). All three behaviours were visually distinguishable. Figure 2 shows that ruminating in particular can be very easily distinguished from the other behaviours due to the associated low overall accelerometer magnitude values, for both ear and collar data. Grazing and non-eating behaviours looked visually similar, although the non-eating activities produced higher overall magnitudes for acceleration compared to grazing for both collar and ear datasets.

Table 3 lists the feature rankings obtained for classifying eating behaviours using either ear or collar data based on the ReliefF algorithm. As illustrated previously, the features were computed on the following signals: accelerometer (A), accelerometer derivative (AD; rate of change), gyroscope (G), gyroscope derivative (GD; rate of change). The rankings of the features differed between ear and collar. The feature: dominant frequency (A) ranked highest for both sensor locations. The feature: zero crossings (A) ranked third for both ear and collar, but the other four out of the six highest ranking features were very different. For the ear, two dominant frequency features (AD and GD) and two gyroscope features—maximum (G) and zero crossings (G)—ranked very high, whereas for the collar, statistical features such as minimum (A), spectral entropy (G), signal area (G) and mean (G) ranked at the top of the list. In general, for both ear and collar data, gyroscope-based features dominated the overall picture with 11 and 10 gyroscope features among the top 15 features for ear and collar, respectively. 

Spectral area (A, AD), and minimum (GD, AD) ranked at the bottom for both sensor positions. Similarly, many of the statistical features for A ranked relatively low, such as kurtosis (A), signal area (A), mean (A), maximum (A) and standard deviation (A).

### 3.2. Assessment of Overall Classification Performance

An initial comparison of the performance of the grazing classification between different learner types can be provided using values of the overall accuracy for both ear and collar mounted sensors. Table 4 reports the maximum overall accuracy value obtained for each type of learning algorithm for both ear and collar data, and how many features out of the 44 were utilised by the model to achieve this maximum accuracy value.

The highest overall accuracy of 92% was obtained for collar data using a random forest classification model with 39 out of the available 44 features. The lowest peak overall accuracy of 67% was obtained for ear data using a support vector machine and four features. Compared to the other learning algorithms, the support vector machine models performed particularly bad, as increasing the number of features beyond four for ear and two for collar did not improve the overall classification performance, whereas the other learning algorithms did benefit from using more than the few highest-ranking ones. AdaBoost and kNN yielded similar results for ear (kNN 79%—22 features, AdaBoost 81%—39 features) and collar (kNN 87%—18 features, AdaBoost 85%—35 features), but both algorithms were outperformed by the random forest model. The average difference between ear and collar accuracy for random forest, kNN and AdaBoost was 4.75%, with collar data generally yielding slightly better results compared than ear data.

**Figure 3:**
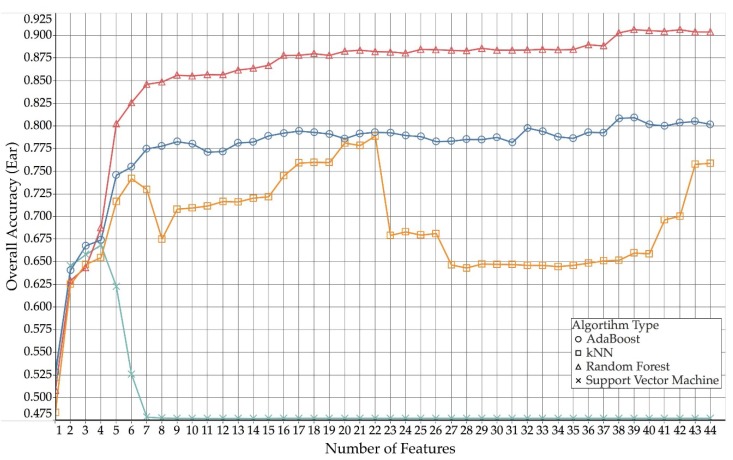
Comparison of overall accuracies for ear, over used number of features (AdaBoost, kNN, random forest and support vector machine).

**Figure 4:**
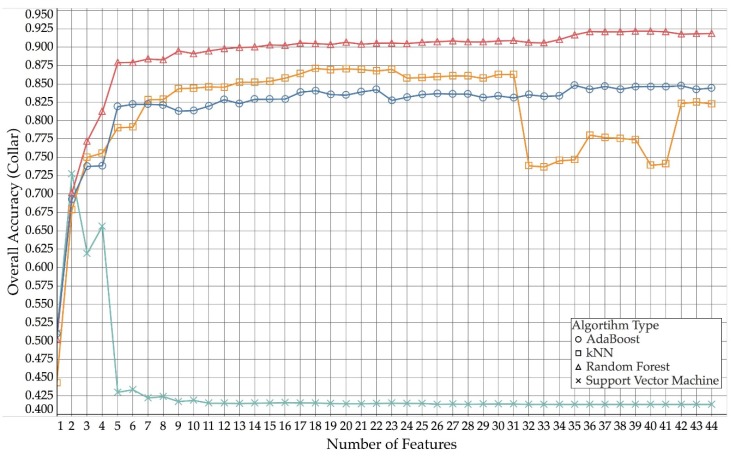
Comparison of overall accuracies for collar, over number of used features (AdaBoost, kNN, random forest and support vector machine).

Figure 3 (collar) and Figure 4 (ear) show how the different algorithm types compare across different numbers of features used for collar and ear data, respectively.

**Figure 5:**
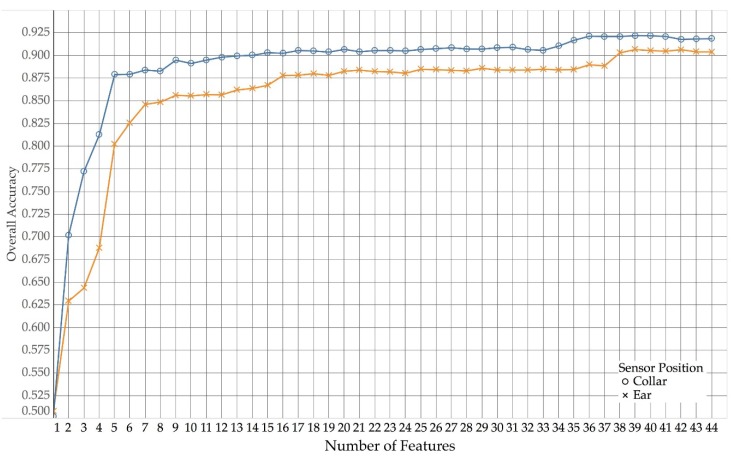
Overall accuracies for ear and collar data over number of features using Random Forest.

Due to random forest models consistently outperforming other algorithm types, we only investigated random forest models in more detail. Figure 5 shows how overall accuracy compares between ear and collar data for random forest models.

As shown in Figure 5, collar data yielded a higher overall accuracy than the use of ear data across all numbers of features. As more and more features were added, the overall accuracy slowly increased. However, for both collar and ear, the trend of increasing accuracy reached a fist plateau at around 16 features (with a first peak around four to six features) and then only improved again, when more than 36 (ear) and 38 (collar) features were used, after which both performance curves reach a second plateau.

### 3.3. Assessment of the Performance of the Classification of Specific Activities

Table 5 shows a confusion matrix produced after running the random forest model using 39 features for collar data, and Table 6 shows the evaluation of performance of the model based on the confusion matrix.

Using collar data and 39 features yielded high performance values across all three classes, with grazing yielding the highest among the three. Precision values across the three behaviour types ranged from 89–96%, Recall from 87–95%, F-score from 89–95% and specificity from 91–98%. This yielded average values of 92.54% for precision, 91.67% for recall, 92.05% for F-score and 95.35% for specificity.

Overall, classification performances for all three behaviour types were very high. Despite non-eating behaviour yielding the lowest precision and specificity and ruminating yielding the lowest performances for recall and F-Score in the above comparison, performance values of 87% and above showed that the behaviours were being classified very well.

Table 7 and Table 8 show confusion matrix and results obtained using random forest with 39 features from the ear dataset.

Similar to the collar results, grazing once again yielded the highest performance values for precision and F-score in the ear data. The best specificity was achieved within the grazing behaviour. Performances were good across all behaviours with precision in the range of 89–95%, recall 86–93%, F-score 88–92% and specificity 89–98%. This yielded average values of 91% for precision, 89.67% for recall, 90.33% for F-score and 94.67% for specificity.

Overall, the classification performance for ear data was very good similar to the classification results for collar data previously observed. For both ear and collar data classification, grazing behaviour yielded the highest values for Precision, F-Score, and Specificity, whilst the non-eating behaviour yielded the highest recall values. Overall, performance values of 86% and above across all behaviours and metrics demonstrated that robust classification of the three behaviours was possible.

Overall, performances obtained for ear were slightly lower than those obtained for collar. However, they were not significantly different. 

## 4. Discussion

To the authors knowledge, this is the first study that compared different classification algorithms, features evaluated from data series, and placement of sensors for the classification of eating behaviours in sheep. Results from this study demonstrated that distinguishing between grazing, ruminating, and non-eating behaviours is possible with high accuracy and little misclassification between the different behaviours. In the current study, overall accuracies of 91% for ear and 92% for collar data were obtained with a random forest classification model utilising 39 out of the 44 available classification features, showing improved classification performance compared to previous studies [2,5,19]. For example, Alvarenga et al. [2] had an overall accuracy for 85% (vs. 92% in the current paper) with no classification of rumination; Marais et al. [19] had grazing misclassified as lying down on 35% occasions; McLennan et al. [5] only distinguished active vs. non-active behaviours, with grazing and ruminating merged together with other active behaviours such as running. One of the reasons for the improved accuracy and classification performance in the current study could be that we used both accelerometer- and gyroscope-based features. None of the previous studies have utilised gyroscope-based features. The fact that gyroscope-based features formed the majority of the top-ranking features in the current study suggested that utilising the additional information obtained from the gyroscope was a key contributor to reliable classification of sheep eating behaviour. A combination of the orientation and angular velocity of the sensor is particularly relevant for eating behaviours that are complex and include jaw movements, such as chewing, regurgitation and swallowing of boluses. Similar results have been found in the classification of more complex human activities, e.g., wrist motion [44]. 

The comparison of classification algorithms, and the ranking of signal-extracted features by their relevance to the classification problem, was another key aspect of this study. There is scant literature in precision livestock comparing different algorithms; however, it has been done in other computation fields [45]. For both ear and collar data, the random forest performed best compared to kNN, SVM and AdaBoost. Random forests [34] has been known to produce the best classification accuracies in different scientific fields [46,47,48]. Moreover, the previous published work on behaviour classification of sheep has also used random forest successfully [2,16]. There are multiple advantages of random forest models—for example, their ability to handle non-linearly correlated data, robustness to noise and being fast and scalable, which make them very popular in various scientific domains. 

With respect to feature selection, in the current study classification accuracy increased very quickly (for the random forest) as more features were added and reached an almost plateau-like performance point after a few features. For the collar, overall accuracy surpassed 87% with only five features. From there, model performance kept increasing as more features were added, but not nearly as quickly as it did over the initial five. Similarly, for ear data, after the first seven features accuracy values surpassed 85% and only slowly increased from there towards the maximum of 91% as more features were added. This suggests that utilising the random forest algorithm as proposed in the current paper with only 5–7 features can still yield highly accurate results. This is of relevance especially for real-time systems as large feature sets are problematic due to computational complexity and have higher storage and/or power requirements [49]. 

One key factor in achieving robust and high-performing classification models is the dataset that is used to train and validate the classification models. In the current study data were collected over a period of eight days, using six different sheep. The availability of ground truth information was limited by the number of video recordings made during that period, but we still had 14 datasets with 27,317 data points in total. This forms a considerably larger dataset than those used in previous literature [2,19]. However, an even larger dataset is likely to further improve the robustness of the model. In addition to the number of samples in the dataset, the fact that the presence of all three classes was balanced in the current dataset unlike previous studies [2,19] also allowed for robust and reliable classification model.

Overall, collar data did yield slightly higher accuracies than ear data (maximum Accuracy: collar 92%, ear 91%), but overall, the differences between collar and ear data were almost negligible. However, with future applications in mind, the ear might be the more practical location for the sensor due to the possibility of integrating the sensor into the ear tag. This has practical benefits over the collar and could be integrated with existing electronic identification system for sheep. However, this needs to be further evaluated and tested. Despite this, the results from this study are promising and could be of significant value for animal health welfare. Changes in the eating behaviour of sheep could be indicative of health or management problems (e.g., quality of pasture) [50]. Furthermore, for such an automatic monitoring device to be commercially viable, it would have to be validated on a larger sample and more advanced machine learning techniques utilizing the quaternion structure of date could be explored [51,52]. In the current study the non-eating behaviours were merged into one category; further studies could analyse a more comprehensive list of behaviours to discriminate them from eating behaviours.

## 5. Conclusions

This study has shown that the behaviours of grazing, ruminating and non-eating can be differentiated with high accuracy using machine learning techniques on data obtained from an accelerometer and gyroscope sensors attached to collars and ear tags on sheep. Classification using collar data yielded slightly better performances than ear data, with overall accuracies of 92% for collar and 91% for ear. Gyroscope-based features were shown to have great importance in the classification of sheep eating behaviours, an aspect that had not been covered by previous studies in the field.

It was shown that random forest models yielded the highest overall accuracies, outperforming a range of other learners used in this study. The optimum number of feature characteristics to be incorporated into the model was 39, for both ear and collar data.

These findings are significant as they confirm that there is potential to classify eating behaviours in sheep with very high accuracy and this could be used to develop a device for automatic monitoring of feed intake in the sheep sector that may help with monitoring animal health and welfare and improving management strategies.

## Figures and Tables

**Table 1:** Ethogram defining eating behaviours used for manual classification of behaviours from video recordings.

Behaviour	Definition
Grazing	Standing or walking with head down, biting, chewing grass or searching for food patches
Ruminating	At rest and ruminating or in the process of regurgitating a bolus
Non-eating behaviour	No jaw movement, sheep could be walking standing or lying

**Table 2:** Percentage of non-mixed and mixed windows.

Type of Sample	Ratio [%]
Non-Mixed	95.55
Mixed	4.45

Percentage of samples that are non-mixed or mixed for the data in this study (16 Hz and 7 s windows).

**Table 3:** Feature rankings and predictive values for collar and ear data using ReliefF. Colours indicate the feature rank from high (blue) to low (red). A: accelerometer, AD: accelerometer derivative (rate of change), G: gyroscope, GD: gyroscope derivative (rate of change).

Type	Name	Rank Ear	Rank Collar
A	Dominant Frequency	1	1
AD	Dominant Frequency	2	28
A	Zero Crossings	3	3
GD	Dominant Frequency	4	22
G	Maximum	5	13
G	Zero Crossings	6	11
G	Mean	7	6
G	Signal Area	8	5
GD	Signal Area	9	7
GD	Mean	10	8
G	Spectral Entropy	11	4
GD	Interquartile Range	12	14
G	Dominant Frequency	13	12
GD	Standard Deviation	14	15
AD	Zero Crossings	15	19
A	Minimum	16	2
G	Standard Deviation	17	21
GD	Maximum	18	20
GD	Zero Crossings	19	26
AD	Signal Area	20	9
AD	Mean	21	10
G	Interquartile Range	22	27
GD	Spectral Area	23	32
AD	Interquartile Range	24	18
AD	Spectral Entropy	25	25
A	Spectral Entropy	26	23
G	Spectral Area	27	40
AD	Standard Deviation	28	16
AD	Maximum	29	24
GD	Spectral Entropy	30	31
GD	Kurtosis	31	33
G	Minimum	32	29
A	Interquartile Range	33	17
A	Kurtosis	34	38
A	Standard Deviation	35	30
A	Maximum	36	34
AD	Kurtosis	37	39
A	Mean	38	35
A	Signal Area	39	36
G	Kurtosis	40	37
AD	Spectral Area	41	41
GD	Minimum	42	43
A	Spectral Area	43	42
AD	Minimum	44	44

**Table 4:** Maximum overall accuracy values of different learner types trained algorithm using all 44 features for each ear and collar data.

Algorithm	Ear		Collar	
	Number of Features	Overall Accuracy	Number of Features	Overall Accuracy
Random Forest	39	91%	39	92%
Support Vector Machine	4	67%	2	73%
*k* Nearest Neighbour	22	79%	18	87%
AdaBoost	39	81%	35	85%

**Table 5:** Confusion matrix using collar data and 39 features to assess the performance of classification of specific eating behavioural activities based on random forest.

	Prediction		
Eating Behaviour	Grazing	Non-Eating Behaviour	Ruminating
Grazing	30.06%	1.65%	0.53%
Non-eating behaviour	0.68%	39.16%	1.55%
Ruminating	0.42%	2.96%	22.98%

**Table 6:** Performance of the classification using collar data and 39 features of specific eating behavioural activities based on random forest.

	Precision	Recall	F-score	Specificity
Grazing	96%	93%	95%	98%
Non-eating behaviour	89%	95%	92%	91%
Ruminating	92%	87%	89%	97%

**Table 7:** Confusion matrix using ear data and 39 features to assess the performance of classification of specific eating behavioural activities based on random forest.

	Prediction		
Eating Behaviour	Grazing	Non-Eating Behaviour	Ruminating
Grazing	27.3%	2.3%	0.3%
Non-eating behaviour	2.6%	43.0%	2.1%
Ruminating	0.4%	3.3%	18.7%

**Table 8:** Performance of the classification using ear data and 39 features of specific eating behavioural activities based on random forest.

	Precision	Recall	F-score	Specificity
Grazing	95%	90%	92%	98%
Non-eating behaviour	89%	93%	91%	89%
Ruminating	89%	86%	88%	97%
